# Conceptualizing Resilience: Impacts of Adverse Stressor Severity, Chronicity, and Relevance

**DOI:** 10.1093/geroni/igaa057.3028

**Published:** 2020-12-16

**Authors:** Michael Griswold, B Gwen Windham, James Henegan, Karen Bandeen-Roche, Matthew Mcmullan, Anna Kucharska-Newton, Priya Palta, Thomas Mosley

**Affiliations:** 1 The University of Mississippi Medical Center, Jackson, Mississippi, United States; 2 University of Mississippi Medical Center, Jackson, Mississippi, United States; 3 Johns Hopkins Bloomberg School of Public Health, Baltimore, Maryland, United States; 4 Gillings School of Global Public Health, University of North Carolina at Chapel Hill, Chapel Hill, North Carolina, United States; 5 Columbia University Irving Medical Center, New York, New York, United States

## Abstract

Guidance on frameworks for physical resilience, resistance and reserve are underdeveloped. We examined different “physical resilience” characterizations within n=6,538 Atherosclerosis Risk in Communities Study participants (median age: 75 years), followed for 5+ years. Fifteen illustrative clinical, lifestyle and social stressors, each having varying levels of severity, chronicity and relevance, were paired with six functional outcome measure trajectories. Contrasts were made against four fundamental comparator groups (including those without stressors). Particular pairings of stressors and functional measures substantially impacted resilience classifications and related determinants. For example, 5-year recurring robustness (0/5 frailty indicators) was only 12% for participants after Heart Failure (HF), versus 47% with no HF event; relative-risk: RR=0.26 (95%CI: 0.15,0.44). Conversely, recurring robustness was 43% after reporting low social support versus 51% with adequate support; RR=0.87(0.73,1.02). We highlight major components that impacted resilience determinations and outline a broad conceptual framework to help optimize physical resilience assessment and aid future research. Part of a symposium sponsored by Epidemiology of Aging Interest Group.

